# Metallic Nanoparticles: A Promising Arsenal against Antimicrobial Resistance—Unraveling Mechanisms and Enhancing Medication Efficacy

**DOI:** 10.3390/ijms241914897

**Published:** 2023-10-04

**Authors:** Shahid Wahab, Alishba Salman, Zaryab Khan, Sadia Khan, Chandran Krishnaraj, Soon-Il Yun

**Affiliations:** 1 Department of Food Science and Technology, College of Agriculture and Life Sciences, Jeonbuk National University, Jeonju 54896, Republic of Korea; shahid@jbnu.ac.kr (S.W.); krishnarajbio@gmail.com (C.K.); 2 Department of Agricultural Convergence Technology, College of Agriculture and Life Science, Jeonbuk National University, Jeonju 54896, Republic of Korea; 3 Nanobiotechnology Laboratory, Department of Biotechnology University of Malakand, Dir Lower, Chakdara 18800, Khyber Pakhtunkhwa, Pakistan; alishbaawais97@gmail.com (A.S.); zaryabtkr@gmail.com (Z.K.); uswah8580@gmail.com (S.K.)

**Keywords:** metallic nanoparticles, antibacterial resistance strains, antifungal resistance strains

## Abstract

The misuse of antibiotics and antimycotics accelerates the emergence of antimicrobial resistance, prompting the need for novel strategies to combat this global issue. Metallic nanoparticles have emerged as effective tools for combating various resistant microbes. Numerous studies have highlighted their potential in addressing antibiotic-resistant fungi and bacterial strains. Understanding the mechanisms of action of these nanoparticles, including iron-oxide, gold, zinc oxide, and silver is a central focus of research within the life science community. Various hypotheses have been proposed regarding how nanoparticles exert their effects. Some suggest direct targeting of microbial cell membranes, while others emphasize the release of ions from nanoparticles. The most compelling proposed antimicrobial mechanism of nanoparticles involves oxidative damage caused by nanoparticles-generated reactive oxygen species. This review aims to consolidate knowledge, discuss the properties and mechanisms of action of metallic nanoparticles, and underscore their potential as alternatives to enhance the efficacy of existing medications against infections caused by antimicrobial-resistant pathogens.

## 1. Introduction

Infectious diseases caused by microbes are a significant global health concern, with an escalating economic burden. The continuous increase in antibiotic resistance rates, which continues to accelerate annually [1], highlights the urgent need for innovative solutions. This concerning trend has resulted in higher mortality and morbidity rates among patients, leading the World Health Organization (WHO) to designate antimicrobial resistance as one of the top three global health challenges [2]. The consequences of antimicrobial resistance extend across various aspects of healthcare, including cancer treatment, premature infant care, transplantation, and surgical procedures. All of these are susceptible to severe disruptions without effective strategies to combat drug-resistant bacteria [3]. The challenges of rising treatment costs, prolonged treatment durations, and increased mortality rates due to antimicrobial resistance require the exploration of effective alternatives [4]. Efforts to address these challenges involve the promotion of diverse novel approaches to regulate antimicrobial medications [5]. These alternatives encompass a range of interventions, including vaccine development, phage therapy, immune stimulants, adjuvants, anti-treatment agents, probiotics, and their various derivatives [6]. Pre-disease vaccination, which aims to prevent diseases by strengthening the human immunological response to bacterial infections, is also a viable strategy. However, widespread implementation faces obstacles related to cost and accessibility [7]. Similarly, strategies like probiotics, prebiotics, symbiotics, and competitive exclusion have been established to deter pathogenic colonization but are constrained by variable efficacy and regulatory processes, such as those imposed by the Food and Drug Administration (FDA) [8]. Furthermore, bacterial cells employ primary defense mechanisms, including enzyme function inhibition and efflux pumps, to reduce susceptibility to antibiotics [9]. Consequently, the era of antibiotics teeters on the brink of extinction, demanding the innovation of new approaches to combat multidrug-resistant strains. Researchers are actively seeking alternative methods to address this pressing issue. In this contemporary context, nano-sized materials emerge as a promising alternative to traditional antimicrobial agents [10]. Nanoparticles (NPs) exhibit distinctive physico-chemical properties, including their controllable small size (ranging from 1 to 100 nm), high reactivity, functionalized structure, and large surface area-to-mass ratio [11]. Leveraging NPs as delivery vehicles for antimicrobial drugs has proven highly effective, mitigating numerous limitations associated with conventional antimicrobial therapies [12]. Notably, microorganisms find it challenging to develop resistance to NPs because these versatile entities can concurrently target multiple cellular pathways [13]. Consequently, NPs have the potential to replace traditional antibiotics and antifungal agents in the treatment of microorganism infections that have become resistant to standard therapies [14]. The synthesis of NPs can be achieved through various methods, each with its own set of advantages and disadvantages. These methods encompass biological, physical, and chemical approaches [15]. Physical methods often yield high quantities of NPs, making them attractive for large-scale production; however, they tend to be energy-intensive and cost-inefficient, which may limit their practicality for some applications [16]. On the other hand, chemical synthesis methods are generally cost-effective and efficient in producing NPs, but they often involve the use of hazardous and volatile chemicals, which can pose environmental risks and safety concerns [17]. In recent years, there has been a growing emphasis on environmentally friendly approaches to NPs synthesis. One such approach is green synthesis, which has garnered significant attention due to its cost-effectiveness, environmental sustainability, and relative simplicity [18]. Green synthesis methods utilize plant extracts, bacteria, and fungi to produce NPs. Among these, plant extracts are particularly desirable as they eliminate the need for complex cell culture maintenance and downstream processing [19]. Harnessing their exceptional chemical properties and physical characteristics, NPs offer a promising avenue for addressing the challenge posed by multidrug-resistant bacteria. In today’s challenging landscape of antibiotic resistance, the need for effective strategies to combat multidrug-resistant strains has reached a critical juncture. This pressing issue necessitates a comprehensive approach that spans research, development, and implementation. Throughout this manuscript, we will delve into the multifaceted nature of effective strategies, exploring their significance in addressing the threat posed by multidrug-resistant strains. From innovative methodologies to strategic interventions, our discussion will emphasize the importance of these strategies as a central theme in our quest to combat this global health crisis. This study will cover various types of metallic NPs, delving into their unique mechanisms of action. It will particularly focus on investigating the antimicrobial properties of different metallic NPs, evaluating how NPs can disrupt multidrug resistance in bacteria and fungi, and assessing their potential as a viable solution for combating bacterial infections.

## 2. Antibiotic Resistance

Antibiotics are used to combat microbial infections, utilizing a range of mechanisms such as inhibiting enzymes, altering membrane structure, and disrupting transcription and translation processes [15]. However, some microbes have evolved to become resistant to antibiotics, posing a significant challenge to the efficacy of antimicrobial agents [20]. Antibiotic resistance is a primary factor contributing to increased drug dosages, extended hospital stays, heightened toxicity, and elevated mortality rates [21]. Multiple factors, such as the over-prescription, misuse, and excessive use of antibiotics, including their extensive use in agriculture, and the scarcity of new antibiotics, all contribute to the occurrence of antibiotic resistance [22].

Antibiotic resistance is a formidable challenge in combating microbial infections caused by bacteria. However, it is important to note that antibiotic resistance is not confined solely to bacteria. Fungi, another category of microorganisms, can also develop mechanisms of resistance to antimicrobial agents [23]. While the primary focus of this section remains on bacterial resistance, the emergence of antifungal resistance is a significant concern in medical and agricultural settings. Fungi, like bacteria, can adapt and develop various strategies to resist the effects of antifungal drugs, posing additional complexities in the fight against microbial infections. Although this review predominantly addresses bacterial antibiotic resistance, it is vital to recognize the broader landscape of antimicrobial challenges, which includes both bacterial and fungal aspects [24].

**Figure 1:**
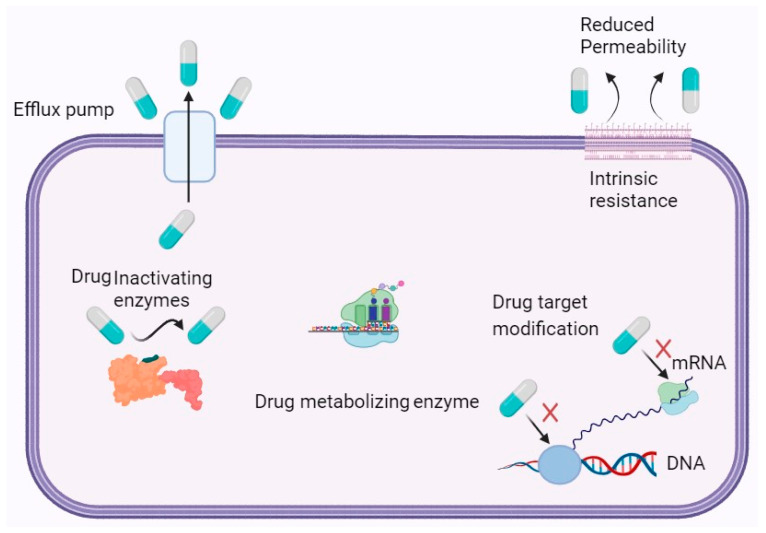
Schematic Illustration of Antibiotic Resistance Mechanisms in Bacteria.

Bacteria employ multiple mechanisms for developing resistance to antibiotics, including intrinsic mechanisms that are typically genetically transmitted. Unlike eukaryotic cells, bacteria are prokaryotes and lack a nuclear membrane to protect their genetic material [25]. As a result, random or spontaneous mutations can occur frequently in the bacterial genome due to the exposed nature of their genetic material. Excessive exposure of a bacterial strain to a particular antibiotic can lead to genetic mutations that give rise to a novel protein that helps the bacterium fight against the antibiotic [26]. The bacterium employs various methods to develop intrinsic resistance to antibiotics, and one of these methods involves utilizing newly produced proteins as tools for survival [27]. Specifically, the protein functions by obstructing the intake of the antibiotic into a bacterial cell, thereby conferring resistance [28]. This involves the production of a mutated protein that triggers the efflux pump present in the bacterial cellular wall. As a result, after antibiotics enter a cell, they are recognized by the altered protein and subsequently pumped out from the cell through an efflux pump [29]. Additionally, bacteria can utilize an enzymatic reaction to inactivate antibiotics as another intrinsic resistance strategy [30]. Bacteria can acquire antibiotic resistance through various mechanisms, such as modifying the antibiotic target, evading the antibiotic target, and vertical gene transfer [31]. The adaptive mechanism employed by bacteria during an infection involves the development of biofilms, which play a crucial role in their survival [32]. Biofilms, complex communities of microorganisms, also interact with their environment. In the case of metalloids and heavy metals, these biofilms can act as both a protective barrier and a source of resistance. They have been found to sequester and immobilize metalloids and heavy metals, reducing their toxicity to the bacteria within the biofilm. These arrangements eventually lead to reduced buildup of antibiotics within the bacteria cells, leading to suboptimal therapeutic levels of the medication [33]. Consequently, higher and more frequent doses of antibiotics may be necessary, which can have dangerous effects on humans and animals. Figure 1 illustrates the mechanism of antibiotic resistance. 

## 3. Mechanistic Action of MNPs against Bacteria

**Figure 2:**
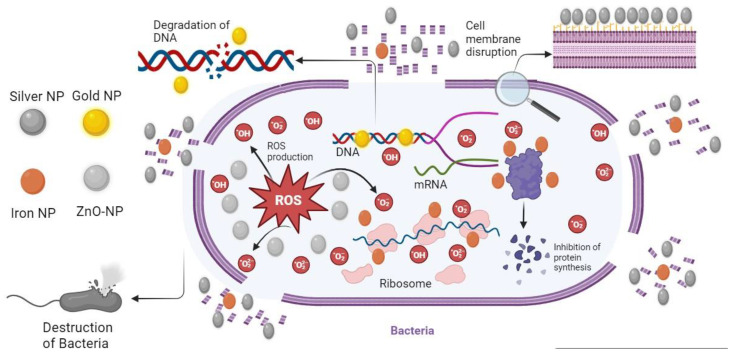
Illustration of the Mechanistic Approach of NPs Against Bacteria.

NPs exhibit unique properties that set them apart from their macroscopic counterparts, making them highly effective in various applications. These properties include crystallinity, exceptional stability, reduced size, the surface plasmon resonance effect, unique shapes, and a higher surface-to-volume ratio [34]. These attributes bestow upon NPs exceptional antibacterial, antifungal, larvicidal, and antiprotozoal capabilities. Specifically, NPs’ distinct size, crystal structure, and reduced dimensions position them as superior alternatives to existing antibiotics, offering potential relief from the burden of antibiotic resistance [35]. Additionally, NPs demonstrate manageable morphologies and excellent size uniformity. Anisotropy, a crucial property of NPs, implies that different facets of their crystal structure possess distinct reactivity. The optical properties of metallic salts transform when converted into nanoforms, enabling significant customization of these characteristics. These remarkable property alterations, combined with the ability to tailor them to specific requirements, establish NPs as a highly promising avenue for addressing antibiotic resistance. Moreover, these changes in properties, coupled with the potential for tailoring them to specific needs, position NPs as a highly promising avenue for addressing antibiotic resistance. Several factors contribute to the antibacterial activity of NPs against bacteria. These include their large surface area that comes into contact with bacteria, electrostatic interactions, or hydrophobic interactions [36]. NPs that interfere with bacteria typically cause oxidative stress, enzyme inhibition, protein degradation, and changes in gene expression [37]. Nevertheless, oxidative stress, metal release, and non-oxidative pathways are the most common antibacterial mechanisms [38]. Among these mechanisms, Reactive Oxygen Species (ROS)-induced oxidative stress stands out as one of the main mechanisms supporting NPs in their antibacterial operation. In bacteria, ROS is produced primarily through aerobic respiration and is balanced by antioxidant cell machinery; however, an excess of ROS can lead to more significant insults, biomolecular oxidation, and cellular damage [39]. Further, when investigating the generation of ROS by NPs, it is crucial to consider the environmental conditions under which this phenomenon occurs. ROS generation by NPs can vary significantly depending on whether it takes place under light or dark conditions [40]. NPs can be viewed as reactive centers, particularly when exposed to electromagnetic activation, such as light conditions, which can greatly influence their intensity and kinetics. Metal ions are slowly released by metal oxides that are carried by the cell to the inner cell, where they interact with protein and nucleic acid functional groups [41]. This interface alters the composition of the holding cell, blocks the enzyme, and intervenes with normal bacteria in the cellular processes [42]. NPs that interfere with the bacteria cell wall create a focal stream of ions that continually emit ions and cause more toxicity to the cells [43]. The surface morphological features of NPs have fundamental effects on their behavior, and where the surface of the NPs is rougher; dissolution happens more rapidly [44]. The association of NPs with the cell wall is implicated in non-oxidative processes. In bacteria, the protective barriers to environmental defense are the cell membrane and cell wall. Different adsorbent ways for NPs are given by the components of the cell membrane and component [45]. The Gram-negative bacterial cell wall comprises phospholipids, lipopolysaccharides, and lipoproteins, forming a barrier only for certain macro molecules to enter [46]. The cell wall consists of a thin layer of peptidoglycans and abundant pores for the Gram-positive bacteria, which allow foreign molecules to penetrate contributing to the binding to proteins and other cellular components covalently that disrupt bacterial cell activity [47]. Lipid polysaccharides on the Gram-negative bacterial cell wall create regions that are negatively charged and attract NPs [48], and because teichoic acid is expressed only in Gram-positive bacterial strains, NPs are spread throughout the phosphate chain. The result is, therefore, more pronounced in Gram-positive bacteria more than that of Gram-negative bacteria [37]. As an example, Zinc oxide NPs (ZnONPs) were synthesized, and their antimicrobial activity against various bacteria was assessed. The results revealed a reliance on the structure and composition of the bacterial cell wall for the antibacterial effects. These NPs exhibited more potent antibacterial activity against Gram-positive bacteria, while specific components appeared to resist the adhesion of ZnONP to bacterial membranes [49]. Figure 2 show the mechanistic approach of NPs against bacteria.

### 3.1. Effect of Silver NPs against Bacteria

Among different types of NPs, silver NPs (AgNPs) stand out as one of the most potent antimicrobial agents [50]. When considering the antimicrobial properties of AgNPs, it is vital to delve into the diverse mechanisms underlying their effectiveness. These mechanisms are influenced not only by NPs size but also by a spectrum of experimental conditions, including dissolution kinetics, pH levels in the medium, solution ionic strength, media composition, and the specific microorganisms or biofilms encountered. One fundamental aspect of AgNPs’ antimicrobial action is their interaction with the microbial cell membrane. AgNPs possess a remarkable ability to disrupt membrane permeability and interfere with cellular respiration [51]. This disruption stems from AgNPs binding to and penetrating the cell membrane, ultimately affecting its integrity. Additionally, AgNPs can engage with thiol groups within microbial cells. This interaction has the potential to trigger the production of ROS, which can disrupt respiratory enzymes, ultimately leading to microbial cell death [51]. For example, the bactericidal effect of AgNPs against *Escherichia coli* has been observed, potentially attributed to the disruption of the proton motive force [52]. The mode of action of AgNPs, although explored over the last few decades, remains a topic of ongoing research and discussion. Some studies suggest that AgNPs kill or inhibit the growth of resistant bacteria by producing Ag^+^ ions. In this scenario, a redox reaction occurs within the bacterial cell when exposed to AgNPs, resulting in the production of silver ions due to their oxidation [53]. These silver ions then alter the macromolecules which lead to the growth inhibition of bacteria. AgNPs tend to affect bacteria directly rather than producing silver ions. It is proposed that AgNPs degrade the cell membrane of bacteria as they are positively charged and have a higher affinity towards the negatively charged peptidoglycan layer of the bacterial cell membrane [54]. It has also been concluded from some research studies that AgNPs react with the DNA of bacteria and control its replication [51]. When AgNPs penetrate through the cell membrane of bacteria, they release silver ions, and these silver ions turn the DNA into its condensed form and alter its replication process [55]. This alteration results in no replication, and cell death occurs as an endpoint of this reaction.

### 3.2. Effect of Zinc Oxide NPs against Bacteria

To combat the problem of antibiotic resistance, ZnONPs have shown promise in different applications, particularly in combating antibiotic resistance [56]. Among the properties of ZnONP, chemical sensing, semiconducting, electric conductivity, and piezoelectric are included [57]. ZnONP exhibits biocompatibility with human cells and demonstrates efficacy as an antimicrobial agent. For instance, *Bacillus subtilis* and *Staphylococcus aureus*, which are Gram-positive bacteria, werefound to be sensitive to ZnONP [58]. *Escherichia coli*, *Pseudomonas aeruginosa*, and *Campylobacter jejuni* are some Gram-negative bacteria found to be influenced by ZnONP [59]. The antibacterial potential of ZnONP depends on their particle size, morphology, and concentration [60]. Various ZnONP structures have significantly different antibacterial activities. ZnONP of rods and wires shape can easily discern bacterial cell walls as compared to spherical shapes [61]. Flower-shaped particles have been proven to be more efficient than both rod and spherical shaped when observed against *Staphylococcus aureus* and *Escherichia coli* [60]. Enhanced antibacterial activity of ZnONP with a larger surface area and increased concentration was reported [62]. ZnONP of smaller sizes have been proven to be comparatively more effective in their antibacterial activities because of their enhanced potential for penetration [63]. The ZnONP activity was also checked against *Staphylococcus aureus* and *Escherichia coli* and effective results were concluded due to their smaller particle size [64]. These size-dependent activities of ZnONP were the same for both Gram-positive and Gram-negative strains [65]. The concentration of ZnONP has noticeable effects on antibacterial activity [66]. A higher concentration of ZnONP can have enhanced antibacterial activity to increase cell death [67]. In a research study, 80 nm-sized ZnONP was employed to combat *Escherichia coli*. The findings revealed that the suppression of bacterial growth was more pronounced at higher concentrations of ZnONP compared to lower concentrations [68].

Studies about mechanisms through which ZnONP kills bacteria and acts as an antibacterial agents are very limited. Various modes of action of ZnONP are concluded from different research studies. Like AgNPs, ZnONP also inhibits the growth of bacteria by generating ROS such as hydrogen peroxides and hydroxyl radicals’ induction. ROS itself is the cause of different antibacterial mechanisms. For example, ZnO localized interaction causes cell wall damage [69]. ZnONPs exhibit another mode of action by altering the cell membrane and permeability of bacteria. Additionally, they function as carriers of zinc ions, releasing these ions through an oxidation reaction once they enter the bacterial cell. These released zinc ions are highly toxic to bacteria, particularly by weakening the mitochondria [70]. ROS alters several macromolecules in the cell. As a result, they express the oxidative stress gene causing growth inhibition and cell death [71]. ZnONPs are used in the food packaging industries for their potential of incorporation into packaging materials as they release NPs against bacteria and prevent foodborne diseases [72]. 

### 3.3. Effect of Gold NPs against Bacteria

Gold NPs (AuNPs) are a comparatively a more unique type of NPs. AuNPs have various clinical applications in the field of nanomedicine against different strains of bacteria [73]. AuNPs synthesis can be achieved through various techniques, including physical methods such as laser ablation, chemical methods involving chloroauric acid, and biological approaches utilizing plants and their extracts. Among these methods, biological approaches, also known as green synthesis, are favored for their reduced environmental impact, cost-effectiveness, and ease of handling [74]. AuNPs hold the potential for conjugation with other antibiotics, antibacterial peptides, and specific antigens [75]. While AuNPs do exhibit antibacterial properties, they may not be as effective against bacteria as AgNPs [76], However, it is worth noting that they can be cytotoxic and genotoxic to animal cells [77]. AuNPs exhibit low toxicity and greater cytocompatibility when compared to AgNPs. Antibacterial activities of AuNPs against both Gram-negative and Gram-positive strains of bacteria have been investigated [78]. For example, a study assessed the effects of these NPs on both *Mycobacterium tuberculosis* and *Escherichia coli*, revealing significant antibacterial activity against Gram-positive *Mycobacterium tuberculosis* and Gram-negative *Escherichia coli* [79]. Research into the antibacterial mechanisms of AuNPs is currently a topic of active investigation. A study reported that AuNPs can interact with the cell membranes of *Escherichia coli*, leading to membrane degradation and growth inhibition. They also observed that the inhibitory effects varied with changes in surface modification agents [80]. AuNPs with similar size and shape caused cell lysis when PAH (Poly-allylamine hydrochloride) was capped, but not in the case of citrate-capped particles [81]. One of the modes of action of AuNPs involves interaction with cellular barriers, such as cell walls and cell membranes [82]. Another study highlighted distinct interactions between AuNPs and the protective barriers of Gram-negative strains when compared to Gram-positive bacteria. In this investigation, it was observed that Au-DAPT-coated gold nanoparticles significantly increased the permeability of *E. coli* membranes by 70%, leading to nucleic acid leakage. In contrast, the impact on *P. aeruginosa* was measured at 42% [83]. AuNPs can also affect bacteria by interacting with various biological substances like DNA and proteins, inhibiting enzyme activity and neutralizing plasmid movement within bacteria [84]. For instance, it was proposed that AuNPs induce DNA fragmentation, ultimately resulting in bacterial cell death [85]. Another study suggested that the photothermal effect of AuNPs is one of their convincing mechanisms, converting infrared light energy into local heat [86]. This heat causes protein denaturation, cell fluid evaporation, and structural breakdown, resulting in bacterial growth inhibition or death. Furthermore, AuNPs can cause a redox imbalance, similar to AgNPs, by generating ROS that induce cell apoptosis and necrosis. They have the potential to disrupt the macromolecules of bacteria, leading to oxidative stress [87]. Despite these findings, further research is needed to fully uncover the antibacterial mode of action of AuNPs.

### 3.4. Effect of Iron NPs against Bacteria

Like other metallic NPs, iron NPs (FeNPs) have demonstrated their antimicrobial efficacy against numerous pathogenic bacteria, suggesting their potential for use in combating microbial infections [88]. FeNPs are significantly cost-effective compared to silver and gold NPs [89], they are also slightly preferred over silver and other NPs as they are less toxic to humans unlike other NPs, especially silver, which can cause cytotoxicity in various human cell lines [90]. FeNPs are considered less toxic and therefore, Ferumoxytol an intravenous Fe_3_O_4_ formulation, was also approved by the FDA as a treatment for iron-deficient patients. Apart from being less toxic, the byproduct of FeO-NPs, which is iron, can be stored by the body [91]. Antibacterial activities of NPs vary from 80–100 nm of semi-crystalline biogenic Fe_3_O_4_ that have been developed from leaf extract of *T. procumbens* and revealed to have bactericidal activity against Gram-negative bacterium *P. aeruginosa* [92]. Similarly, it was reported that Fe_2_O_3_−NPs produced from *Skimmia laureola* exhibit the highest antibacterial activity against *Ralstonia solanacearum* by degrading the cell wall [93]. In another study, it was suggested that rod-shaped FeNPs produced using *Eichhornia crassipes* leaf extract exhibited the highest inhibition against *Pseudomonas fluorescens* and *Staphylococcus aureus* [94]. FeNPs synthesized from *Gardenia jasminoides* and *Lawsonia inermis*, were tested against *Staphylococcus aureus*, and displayed a noticeable increase in the inhibition zone, going from 15 mm to 16 mm [95]. The antimicrobial activity of FeNPs depends on its surface coating. Different NPs have a different surface coating, and this property of NPs seems to play a huge role in their antibacterial efficiency [96]. FeNPs are used as antimicrobial agents and are usually capped with other compounds, such as alginate, for stability purposes because uncapped FeNPs are slightly unstable [97,98]. FeNPs prove their efficacy through the presence of iron, resulting in the production of ROS, such as H_2_O_2_, that can damage the cell membrane once they enter the intracellular space, ultimately leading to bacterial cell death. The bactericidal activity of FeNPs is a result of the oxidative stress caused by FeNPs-generated ROS [99]. Another possible mechanism of FeO-NPs is the damage of DNA through Fenton’s reaction [100]. In Fenton’s reaction, the superoxide anion O- is dismutased to hydrogen peroxide H_2_O_2_, which releases Fe ions (in the Fe^2+^ or Fe^3+^ oxidation state) that can cause direct damage to bacterial DNA, leading to its growth inhibition [101]. Table 1 describes the methods of synthesis and antibacterial effects of different types of metallic NPs.

## 4. Antifungal Activities of Metallic NPs

Fungal species are versatile in adaptation to any environmental conditions [126]. They have the capability of colonizing even in a medium having an extreme or precarious environment and this adaptability results in a variety of problems. Most agricultural practices are found to be affected by resistant fungal species [127]. All the important stages of crop cultivation, such as sowing, growth, production, and after-harvest stages, can be adversely affected. Nowadays chemical treatments are used to control the negative effects of pathogenic fungal species [128]. Despite the low cost and easy availability, the excessive use of chemical suppressors leads to a variety of problems, including environmental pollution, human disease, and ecological imbalance. Additionally, these can also cause resistance in fungal species, hence generating stronger fungal species that cannot be treated with chemical products [129]. Recently, environment-friendly alternatives have been introduced that are used to counter the negative effects of chemical remedies, but they still have considerable limitations. Plant extracts and essential oils can be used alternatively, but they can be processing intensive, and their high acquisition cost and persistent application make them a less attractive approach [130]. However, the incredible potential of NPs can be exploited against resistant fungal species [131] as they are proved effective and applicable as opposed to bacteria that are resistant as mentioned in the above section. 

## 5. Mechanism of Action of Metallic NPs against Fungal Species

**Figure 3:**
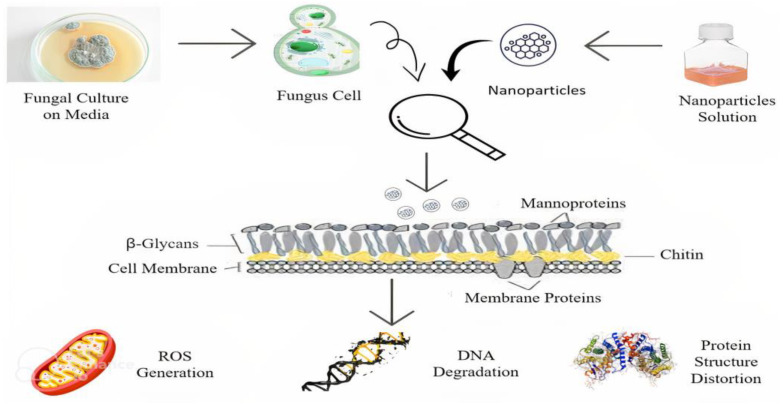
Illustration of NPs Antifungal Mechanism.

Metallic nanoparticles [MNPs] have various interactions with fungi that can result in advantageous or detrimental outcomes, depending on factors such as the NPs type, fungal species, and environmental conditions [132]. Many NPs have been reported to act in several ways against resistant fungal species. Exposure to NPs induces changes in the fungal cell wall, including surface alterations, cell aggregation, pit and pore formation, and overall deformation [133]. Studies have revealed that NPs may directly interact with and embed themselves within fungal cell walls during adsorption, resulting in morphological changes [134]. The inner membranes also undergo distortion, with altered organelle arrangement, such as an increased count of intracellular vesicles and vacuoles, and reduced cytoplasmic content, causing the release of cellular contents [135]. Smaller NPs may facilitate fluid-phase endocytosis, bypassing the need for significant cell wall damage. Exposure to NPs can result in alterations in gene expression and protein levels. Once NPs are inside the cell, some can intercalate with nucleic acids intracellularly [136]. Furthermore, some studies have indicated that ions are more toxic than their NP counterparts, possibly due to their size, which facilitates penetration into cells, or their ability to complex with other biomolecules such as proteins, nucleic acids, and negatively charged lipids [137]. NPs can profoundly impact fungal hyphae and spores. Exposure to NPs has been shown to deform hyphae, causing them to appear distorted and shrunken [138]. NPs alter growth patterns, leading to clumping and thinning of hyphal fibers. NPs can inhibit the formation of biofilms, as hyphae development is essential for biofilm formation and adherence, which are required for pathogenesis and colonization. The inhibition of filamentation is primarily driven by cell wall disruption. NPs can also affect pre-formed biofilms and deposit onto extracellular polysaccharides (EPS), crucial for structural integrity [139]. Additionally, MNPs can stimulate the production of ROS within fungal cells, leading to oxidative stress and cellular damage [140]. Figure 3 Showed the antifungal mechanism of NPs’.

### 5.1. Effect of AgNPs against Fungus

AgNPs are thoroughly studied in various scientific fields [141]. The antimicrobial, antioxidant, and anticancer properties of AgNPs, along with their low cost and ease of production, indeed make them appealing to therapeutic agents. However, there is some disagreement regarding their biocompatibility and toxicity. While AgNPs have been utilized and researched extensively against various microorganisms and fungi [142], previous studies showed that several AgNPs have reasonable activities against phytopathogenic fungi [143]. Ag^+^ ions and AgNPs can modify fungal cell transcriptomes, epigenomes, and metabolomes, leading to crucial functional alterations. This involves the down-regulation of genes related to the tricarboxylic acid cycle, redox metabolism, ergosterol synthesis, and lipid metabolism, ultimately causing structural modifications, primarily in fungal cell membranes [144]. AgNPs have been shown to have powerful antifungal properties based on their structural characteristics [145]. Mostly spherically and small-sized NPs are found to have a potential role against the different phytopathogenic fungal strains [146]. Sizes ranging from 10 to 30 nm have been found to have relatively effective antifungal activities [147]. Due to their small size, AgNPs can easily penetrate cell membranes, and their toxicity is partly linked to the production of ROS. This leads to the unification of fungal hyphae and mycelium, effectively deactivating these pathogens [148]. Alternatively, 40 to 70 nm NPs showed potent inhibitory activities by destroying mycelium and fungal spores, resulting in significant membrane rupture [147]. The concentration of NPs applied is a critical factor in fungal-NP interactions, with a significant impact on fungal strains. AgNPs attach to the fungal surface via electrostatic attraction. As AgNPs accumulate outside the cell, they release Ag^+^ ions, which enter the cell and neutralize or incapacitate these pathogens [149,150]. To determine the optimal concentration for effective antifungal activity, previous studies have explored various concentrations [151,152]. Surprisingly, lower concentrations have often demonstrated greater potency compared to higher concentrations. For example, AgNPs at a concentration of 20 ppm, produced from extracts of *Psidium guajava* and *Momordica charantia*, inhibited the growth of fungal strains including *Fusarium oxysporum*, *Aspergillus flavus*, and *Aspergillus niger* [153]. Similarly, research revealed that a 25-ppm concentration of AgNPs synthesized from extracts of *Trichoderma viride* completely inhibited the growth of *Alternaria solani* [154]. In another study, different concentrations of AgNPs (10, 25, 50, and 100 ppm) synthesized from green and black tea were tested against *Aspergillus parasiticus*, with maximum inhibition noted at a concentration of 100 ppm [155].

### 5.2. Effect of Copper NPs against Fungus

Copper nanoparticles (CuNPs) are known for their substantial antiseptic properties and cost-effectiveness [156]. CuNPs are employed as antimicrobial agents, benefiting from their substantial surface-to-volume ratio, which enables interactions with other particles, enhancing antimicrobial efficacy. Frequently, they are protected by polymers or surfactants to prevent oxidation. Chitosan-coupled CuNPs represent a highly promising nanocomposite, demonstrating remarkable antifungal activity against phytopathogens [157,158]. CuNPs function as fungicides by generating highly reactive hydroxyl radicals (•OH), which have the potential to inflict damage on biological macromolecules like the DNA of fungal pathogens [159]. While numerous studies have highlighted the potential of Cu NPs as effective antifungal agents, compared to research on other MNPs, there is a limited number of studies elucidating the antimicrobial mechanism of CuNPs. It has been suggested that the antimicrobial mechanism of CuNPs shares similarities with that of AgNPs; further research is required to fully elucidate their precise antifungal mechanisms and potential applications [160,161]. The efficacy of CuNPs against microbes depends significantly on various factors, including particle size, morphology, and concentration [162]. There is a huge diversity in size and antifungal activity. The variety of sizes of NPs gives a different extent of antifungal activity, making it difficult to evaluate the exact size of the particles that is the most effective in action [41]. Small size NPs may breach the cellular membrane, causing the leakage of the cellular contents [163]. In terms of shape, mostly spherical NPs have the most potent antifungal properties [164]. Other shapes that are found to show activities against the fungal spores are truncated octahedral, which is highly effective against *Fusarium oxysporum*, *Fusarium solani*, and *Neofusicoccum* sp. The faceted shape was also found to have reasonable activities against *Fusarium oxysporum*, and *Fusarium solani* [147]. To evaluate the optimum concentration for the CuNPs, low, medium, and high concentrations were applied to the population of phytopathogenic fungi. To check the effect, lower concentrations of 0.1, 0.25, and 0.5 ppm of the CuNPs were applied. The 0.1 ppm concentration appeared to promote hard oxidative stress inside the mycelium, while 0.5 ppm concentration was found to have antifungal activities against *Fusarium oxysporum* [165]. In medium concentrations, 5, 10, and 20 ppm of CuNPs were applied to the culture of the *Phytopthora capsici* and *Fusarium oxysporum*. On the third day of the application, antifungal activities were noted. The 5-ppm concentration applied against the fungi showed 49% inhibition of the culture, while 20 ppm of CuNPs was found to prevent 63% of the phytopathogenic fungus [161]. Another study applied CuNPs at 35, 25, 15, and 5 ppm concentrations against *Sparassis crispa*, *Phytophthora cactorum*, *Grifola frondose*, *Megaloceros giganteus*, *Fusarium redolens*, *Fasciola hepatica*, and *Megaloceros giganteus*. Among the applied concentrations, the 35 ppm concentration was found to be the most potent that was able to completely inhibit the development of plant pathogenic fungi [166]. The maximum concentrations of 300, 380, and 450 ppm showed excellent antifungal activities against *Fusarium oxysporum* with maximum antifungal activity noted at 450 ppm concentration [167]. In another study, 50, 100, 500, and 1000 ppm concentrations were applied against *Alternaria alternata*, *Botrytis cinerea*, *Colletotrichum gloeosporioides*, *Monilinia fructicola*, *Fusarium solani*, *Fusarium oxysporum*, and *Verticillium dahlia*. All the phytopathogenic fungi were found to be inhibited at 1000 ppm concentration of CuNPs [168].

### 5.3. Effect of Selenium NPs against Fungus

Selenium nanoparticles (SeNPs) possess broad biomedical applications, serving as antibacterial, antifungal, antioxidant, and anticancer agents, and their biologically synthesized variants demonstrate enhanced compatibility with human tissues. With their size, shape, and synthesis methods being actively investigated for their utility in biological systems, owing to their biocompatibility, low toxicity, and high bioavailability, which render them increasingly valuable in diverse biomedical contexts [169]. For example, biogenically synthesized SeNPs, produced by *Ralstonia eutropha* with a size range of 40–120 nm, exhibited inhibitory effects on the growth of the fungus *Aspergillus clavatus* at a concentration of 500 µg/mL [170]. SeNPs, produced through *Bacillus thuringiensis* with an average size of 50 to 200 nm, exhibited antifungal properties against *Malassezia* and *Aspergillus* by inhibiting spore germination [171]. Studies have evaluated the fungicidal activity of SeNPs synthesized within a size range varying from 50 to 400 nm. These NPs were employed to prevent the formation of *Candida albicans* biofilms. SeNPs exhibited a strong adherence to biofilm, enabling penetration into the pathogenic agents and causing structural damage through sulfur substitution [172]. Trichoderma-mediated SeNPs were tested against *Sclerospora graminicola* in doses ranging from 0 to 1000 ppm. Six different strains of *Trichoderma* spp., including *Trichoderma harzianum*, *Trichoderma virens*, *Trichoderma asperellum*, *Trichoderma longibrachiatum*, *and Trichoderma brevicompactum* were used. *Trichoderma asperellum* showed the efficient synthesis of SeNPs in the form of culture filtrate in the context of fungicidal capacity [173]. SeNPs synthesized through *Trichoderma viride* following a biological method were applied in vitro at various concentrations (50, 100, 200, 300, 400, 500, 600, 700, and 800 ppm) on the plant, and then treated with *Alternaria solani*. Results demonstrated that fungal growth was inhibited by SeNPs at 800 ppm [154]. Yet, inanother study, different concentrations of chemically synthesized SeNPs were evaluated, including 0.1, 0.5, 1, 5, 10, 50, and 100 ppm, against *Macrophomina phaseolina*, *Sclerotinia sclerotiorum*, and *Diaporthe longicolla*. At 10 ppm and above, SeNPs inhibited *Diaporthe longicolla*, and at 50 and 100 ppm they showed activity against *Macrophomina phaseolina. Sclerotinia sclerotiorum* grew and developed unhindered under different concentrations of SeNPs [152]. 

### 5.4. Effect of FeNPs against Fungus

FeNPs have the potential to induce microbial toxicity through a series of interactions, including membrane depolarization, which compromises cell integrity [174], the generation of ROS resulting in lipid peroxidation and DNA damage, and the release of metal ions that disrupt cellular homeostasis and protein coordination [175]. Due to their unique properties, such as biocompatibility, stability, and magnetic characteristics, biomedical fields are increasingly interested in FeNPs, making them promising candidates for applications in antibacterial, antifungal, and anticancer contexts [176,177]. FeNPs were found to inhibit spore germination, substantially reduce mycelium proliferation, and limit oxygen supply for respiration due to their higher surface-to-volume ratio, resulting in the complete coating of fungal microorganisms [178]. FeNPs can be synthesized using plant extracts, enabling the production of a large quantity of antimicrobial agents. As an example, the sensitivity of phytopathogenic fungi to FeNPs synthesized using green and black tea leaves was tested. Various concentrations of FeNPs at 10, 25, 50, and 100 ppm were used against fungi *Aspergillus flavus* and *Aspergillus parasiticus* in vitro. The results showed inhibition activity of 43.5% with FeNPs synthesized in green tea leaf extract and 51.6% inhibition activity with those synthesized in black tea leaf extract both at a dose of 100 ppm [155]. In another study, Fe_2_O_3_ particles with sizes ranging from 10 to 30 nm using a green approach were tested for their fungicide efficacy in opposition to *Alternaria alternata*, *Cladosporium herbarum*, *Trichothecium roseum*, *Penicillium chrysogenum*, and *Aspergillus niger*. It was noted that Fe_2_O_3_ significantly reduced the growth of all the fungal infections that were examined. *Trichotherma roseum* and *Cladosporium herbarum* were determined to be inhibited by 87.74% and 84.89% through the highest levels of spore germination inhibition. *Penicillium chrysogenum* had the maximum inhibitory zone (28.67 mm) caused by iron oxide NPs, followed by the *Aspergillus niger* (26.33 mm), *Trichotherma roseum* (22.67 mm), *Alternaria alternata* (21.33 mm), and *Cladosporium herbarum* (18.00 mm) [178]. Table 2 describes methods of synthesis and antifungal effects of different types of metallic NPs.

## 6. NMs with Antibiotics against Antimicrobial Resistance

In conjunction with antibiotics, NMs display a high degree of antibacterial activity to prevent bacteria from displaying tolerance to different antibiotics [43]. This concludes that NPs synthesis represents the best cure for enhanced bacterial antibiotic resistance [191]. NMs, combined with antibiotics or other antimicrobial agents, can overcome the limitations in their antibacterial potentials when they are used separately. The antibacterial effects of NMs can be improved by combining different antimicrobial agents with them, facilitating their intracellular targeting and improving their drug stabilization [192]. For example, there were combined effects of cephalexin antibiotics plus AgNPs for their improved antibiotic activity and antibacterial activity against *S. aureus* [193]. Another study showed that enhanced antibacterial activity in AgNPs conjugated with streptomycin was reported for *S. aureus* and *E. coli* bacteria [194]. The antimicrobial potential of ZnONP has been well explored in recent decades, although very limited literature is available on its synergistic effects with antibiotics. In the investigation of ZnO-NP, 9AA-HCl, and their conjugates’ effectiveness against *E. coli*, it was noted that the bacterial cell killing achieved by ZnO-NP-9AA-HCl was nearly 100%, a significant improvement compared to their individual use [195]. Microdilution was employed to assess the minimal inhibitory concentration (MIC) for ZnO NPs and various antibiotics (meropenem, ciprofloxacin, and colistin). The MIC values ranged from 2000 to 4000 μg/mL against *P. aeruginosa* when considering the combination of antibiotics and ZnO NPs conjugation [196].

An enhancement in the stability, selectivity, or functionality of antibiotics is the major benefit when they get attached to NPs [197]. Conjugating antibiotics with AuNPs is one of the strategies to improve the potency of the current antibiotic treatment [198]. Also, the conjugated NPs will target the drug in a way that other systematic compounds will not [199]. Different studies reveal that when antibiotics are conjugated with AuNPs, they show enhanced antimicrobial activity compared to that of antibiotics alone [200]. According to the report, antibiotics loaded onto Au-NPs exhibit greater efficacy against both Gram-positive and Gram-negative bacteria compared to the same dose of antibiotics used alone [201]. Loading of drugs to NPs has been done for many antibiotics like ciprofloxacin, neomycin, 5-fluorouracil (anticancer compound), ampicillin, kanamycin, gentamycin, and streptomycin. In a study, vancomycin was conjugated to Au-NPs for the destruction of bacteria resistant to vancomycin, i.e., *Enterococcus faecium* and *Enterococcus faecalis*. The results of the study have shown a 50-fold increase in the antibacterial activity of vancomycin [202]. FeNPs are less toxic and have many applications in the formation of bioproducts [203]. When antibiotics are attached to FeNPs, their constancy, and capabilities increase [204]. FeNPs also act as nanovehicles for carrying antibiotics because iron is very important for bacterial cell viability. For example, when FeNPs were combined with erythromycin, researchers observed enhanced antibacterial activity of erythromycin against *Streptococcus pneumonia*. This indicated that FeNPs acted as nanovehicles for erythromycin delivery [205]. Combining FeNPs with antibiotics reduced the required drug dose [206]. FeNPs conjugates also reduce toxicity of the NPs by enhancing intracellular targeting ability. The inhibitory action of FeNPs and cephalexin conjugation against several types of multidrug-resistant bacteria (*E. coli*, *Bacillus* sp., *S. aureus*, and *Salmonella* sp.) showed that FeNPs conjugated with antibiotics gave zone of inhibition greater than cephalexin alone [207].

## 7. Current Challenges and Future Perspectives

The role of nanotechnology in different areas such as medicine, vaccination, diagnostics, etc. is noticeable [208]. However, the potential of antibacterial and antifungal applications of NMs is currently limited by certain challenges [209]. If the life science research communities manage to overcome these challenges, the applicability and effectiveness of NMs could then help efficiently restore the lost activity of antimicrobials. The potential toxicity of NMs is one of the biggest challenges nanotechnologies are currently facing [210]. NMs are not just potentially toxic to humans but also to the environment. As most NMs are metallic, unfortunately, their toxicity is not well understood [211]. Metallic NMs are very carcinogenic and therefore, using them in immunodeficient patients can result in adverse effects. The possible accumulation of NMs in patients’ bodies can result in human health compromise [212,213]. Therefore, the toxicity of NMs needs to be minimized. NMs, especially physically and chemically synthesized NMs, have adverse environmental effects [11]. The inappropriate disposal of NMs can cause various forms of environmental pollution as they are not easily degraded. Therefore, these NMs get suspended in the air and can travel long distances [214]. When NMs encounter the body, they easily penetrate the skin due to their nanoscale sizes [215]. NMs have harmful effects on air, soil, and groundwater [216]. There is limited literature available regarding the potential risks associated with NMs. The use of NMs that are not well-understood could potentially lead to adverse health effects when employed for therapeutic purposes in immunodeficient patients [217]. This challenge can be addressed through global knowledge-sharing and collaborative efforts. Researchers, nanotechnology experts, and various research organizations from around the world can collectively focus on advancing this research field, acknowledging that it is still in its developmental stages. This collaborative approach holds the potential to enhance the antimicrobial effectiveness of NMs without adverse consequences. 

## 8. Conclusions

The widespread use of antimicrobials has given rise to a challenging global problem: the development of antimicrobial-resistant pathogens, which has become a worldwide issue. Although various strategies are available to combat these AMR pathogens, the problem persists due to their labor-intensive nature and the significant environmental concerns associated with their implementation. Nanotechnology offers a promising alternative to these conventional strategies, presenting a distinct advantage. Two major distinct strategies for NP synthesis exist: green synthesis and chemical synthesis. Among these, green synthesis stands out as a better alternative that addresses environmental concerns. This type of NP synthesis involves plants, which are integral parts of the environment, providing a strong basis for mitigating the environmental concerns associated with chemical strategies. Furthermore, the production of NPs using plants is less labor-intensive and requires a smaller workforce. Another crucial aspect of this alternative is its superiority over other conventional strategies for tackling AMR. NPs demonstrate remarkable effectiveness against AMR pathogens, irrespective of the medium, their structural characteristics, colony size, or other defensive mechanisms that microbes develop over time and exposure to specific treatments. Notably, microbes cannot develop mechanisms to counteract the action of NPs. Nanotechnology has emerged as a viable solution to the global challenge of antibacterial and antifungal resistance. Nevertheless, further experimental support and systematic clinical trials are required to fully elucidate the precise mechanism underlying the antimicrobial characteristics of MNPs.

## Figures and Tables

**Table 1:** Describes the methods of synthesis and antibacterial effects of different types of metallic NPs.

S. No	NPs	Synthesis Method	Bacterial Strains	Summary of Results	References
1	Silver	Justicia *adhatoda* L. leaves	*Pseudomonas aeruginosa*	Inhibit bacterial growth	[102]
2	Gold	Cashew nutshell extract *Anacardim occidentale*	*Pseudomons fluorescens* *Aeromonas bestiarum*	For *Aermonas bestiarum*: MIC values = 294 ± 12.8 μg/mL MBC values = 363 ± 16.2 μg/mL For *Pseudomonas fluorescens*: MIC values = 386 ± 12.7 μg/mL MBC values = 294 ± 9.42 μg/mL	[103]
3	Silver	Leaves extract of *Urtica dioica* (Linn.)	*Bacillus cereus*, *Bacillus subtilis*	For *Bacillus cereus*: MIC values = 284 ± 12.6 μg/mL MBC values = 361 ± 15.7 μg/mL For *Bacillus subtilis*: MIC values = 376 ± 12.5 μg/mL MBC values = 282 ± 9.43 μg/mL	[104]
4	Silver	Banana peel extract	*Bacillus subtilis*, *Staphylococcus aureus*	MICs were 1.70, 5.1, 6.8, and 3.4 μg/mL MBCs value of AuNPs is 0.2, 10.2, 5.1 μg/mL	[105]
5	Gold	From *Trianthema decandra*	*Staphylococcs aureus*, *Streptococcus faecalis*	For *Staphylococcs aureus*: MIC values = 113 ± 9.1 μg/mL MBC values = 111 ± 13.5 μg/mL For *Streptococcus faecalis*: MIC values = 245 ± 12.2 μg/mL MBC values = 176 ± 12.9 μg/mL	[106]
6	Gold	*Citrullus lanatus* rind	*Escherichia coli*, *Bacillus cereus*	For *Bacillus cereus:* MIC value = 50 μg/mL MBC value = 100 μg/mL For *Escherichia coli*: MIC value = 50 μg/mL	[107]
7	Gold	*Agaricus bisporus* extracts	*Staphylococcus aureus*, *Escherichia coli*	MIC values = 50.99 μg/mL for *E. coli* MIC values = 198.2 μg/mL for *S. aureus*	[108]
8	Gold	Using aqueous *Plumeria alba* flower extract	*Escherichia coli*	MIC value = 400 μg/mL	[109]
9	Gold	*Salix alba*	*Klebsiella pneumoniae*, *Bacillus subtilis*, *Staphylococcus aureus*	-----	[110]
10	Gold	*Brassica oleracea*	*Staphylococcus aureus*, *Klebsiella pneumoniae*	MIC values = 25 μg/mL for *S. aureus* MIC values = 50 μg/mL for *K. pneumoniae*	[111]
11	Zinc oxide	*Hibiscus subdarifa* leaf extract	*Escherichia coli*, *Staphylococucs aureus*	For *Escherichia coli* with PZN60: MIC value = 24 ± 1 μg/mL MBC value = 50 ± 1 μg/mL For *Staphylococcs aureus* with PZN60: MIC value = 50 ± 1 μg/mL MBC value = 50 ± 1 μg/mL	[112]
12	Zinc oxide	*Parthenium hysterophorus* extract	*Staphylococcus aureus*, *Bacillus subtilis*	MIC value = 11 ± 0.28 μg/mL for *Staphylococcus aureus* MIC value = 10 ± 0.16 μg/mL for *Bacillus subtilis*	[113]
13	Zinc oxide	*Camellia sinensis* extracts	*Klebsiella pneumoniae*, *Pseudomonas aeruginosa*, *Escherichia coli*	MIC value = 10.3 ± 0.57 μg/mL for *K. pneumoniae* MIC value = 3.3 ± 0.57 μg/mL for *P. aeruginosa*	[114]
14	Iron	*Moringa oleifera* extracts	*Escherichia coli*	MIC value = 59 ± 1.22 μg/mL for *Escherichia coli*	[115]
15	Iron oxide	*Cynometra ramiflora*	*Escherichia coli*, *S. epidermidis*	--------	[116]
16	Iron oxide	*Lagenaria siceraria* leaves extract	*Escherichia coli*, *Staphylococcus aureus*	--------	[117]
17	Gold	Reduction of tetra chloroauric acid with sodium nitrate	*E. coli k12*	MIC value = 7.4 μg/mL	[118]
18	Gold	*M. piperita*	*E. coli*, *S. aureus*	Showed activity against *E. coli* and no activity against *S. aureus*	[119]
19	Gold	Purchased	*Salmonella typhi*, *Salmonella enteritis*	MIC values = 2.5–5 μg/mL	[120]
20	Iron oxide	Chemically by laser ablation in liquid	*Serratia marcescens*, *Escherichia coli*, *Pseudomonas aeruginosa*, *Staphylococcus aureus*	The disc diffusion method found iron oxide NPs inhibitory zones against Gram-negative and Gram-positive bacteria.	[121]
21	Iron oxide	Synthesized from ferric chloride and ferrous chloride using the co-precipitation method	*B. cereus*, *Klebsiella pneumoniae*	At 40 μg/well concentration of Fe_3_O_4_-NPs, the inhibitory zone was 15 mm against *K. pneumoniae* and 13 mm against *B. cereus* At 80 μg/well concentration of Fe_3_O_4_-NPs, the inhibitory zone was 26 mm against *K. pneumoniae* and 22 mm against *B. cereus*	[122]
22	Iron	*Aloe vera* leaves	*Proteus mirabilis*, *Escherichia coli*, *Klebsiella pneumoniae*, *Pseudomonas aeruginosa*, *Shigella flexneri*, *Serratia marcescenes*, *Salmonella typhi*, *Enterococcus faecalis*, *Staphylococcus aureus*	Zone of inhibition (mm) at 40 µg/well: *Escherichia coli* = 15 ± 0.11 *Proteus mirabilis* = 16 ± 0.21 *Klebsiella pneumoniae* = 17 ± 0.54 *Pseudomonas aeruginosa* = 16 ± 0.29 *Shigella flexneri* = 14 ± 0.61 *Serratia marcescenes* = 15 ± 0.58 *Salmonella typhi* = 16 ± 0.66 *Enterococcus faecalis* = 15 ± 0.13 *Staphylococcus aureus* = 15 ± 0.79	[123]
23	Zinc oxide	Synthesized from zinc acetylacetonate hydrate and oleylamine	*E. coli*	Showed excellent antibacterial activity (10 CFU/mL)	[124]
24	Zinc oxide	From leaves and fruits of *C. procera*	*E. coli*, *Vibrio cholerae*	MIC value = 1.6 × 10^5^ − 1.2 × 10^6^ per mL	[125]
25	Silver	Synthesized from *C. procera* leaves and fruits	*Vibrio cholerae*, *E. coli*	MIC value = 5 × 10^6^ − 1.2 × 10^7^ per mL	[125]

**Table 2:** Describe methods of synthesis and antifungal effects of different types of metallic NPs.

S. No	NPs	Synthesis Method	Fungal Strains	Summary of Results and Antifungal Potency	References
1.	Silver	Using PVP as a reducing agent	*Saccharomyces cerevisiae*, *Candida albicans*.	MIC 50 = 0.5 mg/mL and 4 mg/mL against *Saccharomyces cerevisiae* and *Candida albicans*, respectively.	[179]
2.	Gold	Stainless steel (reducing agent) mediated reduction	*Candida albicans* (ATCC 10231)	Cell viability count through the Neubauer chamber gave antifungal activity at 20 mg/mL.	[180]
3.	Gold	For reducing agent citrate are used.	*Candida albicans* (ATCC 10231)	Cell viability count through the Neubauer chamber gave antifungal activity at 40 mg/mL.	[180]
4.	Zinc oxide	Mycological synthesis	*Aspergillus niger*, *Aspergillus fumigatus*, *Aspergillus aculeatus*	A large zone of clearance was obtained with the largest against *Aspergillus fumigatus.*	[181]
5.	Silver	Reduced with ribose and stabilized with sodium dodecyl	*Candida albicans* and *Candida tropicalis*	A high antifungal activity like that of amphotericin B disc.	[182]
6.	Gold	Solvothermal method	*Candida* isolates	The zone of clearance observed to be 4.2 mm/mg against *Candida albicans* & 1.1 mm/mg against *Candida glabrata.*	[183]
7.	Zinc oxide	Use of leaf extracts of medicinal plants such as *Beta vulgaris*, *Cinnamomum verum*, *Cinnamomum tamala*, and *Brassica oleracea var. Italica*	*Candida albicans* and *Aspergillus niger*	*Beta vulgaris* based NPs revealed potency against *A. niger*, *Cinnamomum tamala* based NPs showed activity against *C. Albicans*. Both fungal strains were sensitive to ZnONPsynthesized from *Brassica oleracea var. italic.*	[184]
8.	Silver	*Aspergillus niger* fungal isolates	*Aspergillus flavus*, *Fusarium oxysporum* and *Penicillium digitatum.*	The lower MIC values i.e., 6.75 ± 0.24, 7.45 ± 0.18, and 9.62 ± 0.14 obtained for *Penicillium digitatum*, *Aspergillus flavus*, and *Fusarium oxysporum*, respectively.	[185]
9.	Copper	The use of Cetyl Trimethyl Ammonium Bromide and isopropyl alcohol as reducing agent in the chemical reduction of Cu^2+^	*Curvularia lunata Phoma destructiva Alternaria alternata* and *Fusarium oxysporum.*	The inhibitory zone was 22 ± 1 mm against *Phoma destructiva*, 21 ± 0.5 mm against *Curvularia lunata*, 18 ± 1.1 mm against *Alternaria alternata*, and against *Fusarium oxysporum* was 24 ± 0.5 mm.	[186]
10.	Copper	Extracellular synthesis by *Streptomyces griseus*	Red-root rot disease-causing fungus	52.7% of the disease was reduced with the application of 2.5 ppm CuNPs in the selected bushes.	[187]
11.	Copper	Using CTAB as a reducing agent in chemical reduction method	*Fusarium* sp.	93.98% of fungal growth was inhibited with the application of 450 ppm of CuNPs after 9 days of incubation	[167]
12.	Photo-activated Zinc oxide	Obtained from Alfa Aesar (NanoShield, Germany)	*Botrytis cinerea*	ZnONPs were revealed to cause morphological changes to fungus after treatment with photoinactivation (58%) and NPs at a concentration of 5 × 10^−3^ M.	[188]
13.	Zinc oxide	Alfa Aesar (Ward Hill, MA, USA)	*Penicillium* *expansum* and *Botrytis cinerea*	Results showed that 3 mmoL/L of ZnO NPs can pointedly stop the growth of *P. expansum* and *B. cinerea.*	[189]
14.	Zinc oxide	Ultrasonic method	*Candida albicans*	The minimum concentration of ZnO required to effectively stop the growth of *Candida albicans* was found to be 0.1 mg/mL, resulting in more than 95% inhibition	[190]

## Data Availability

Not applicable.
